# Proton dose perturbations caused by high‐voltage leads from implanted cardioverter defibrillators

**DOI:** 10.1120/jacmp.v13i4.3813

**Published:** 2012-05-10

**Authors:** Landon S. Wootton, Jerimy C. Polf, Stephen Peterson, Jeff Wilkinson, Marc A. Rozner, Peter A. Balter, Sam Beddar

**Affiliations:** ^1^ Department of Radiation Physics The University of Texas MD Anderson Cancer Center Houston Texas; ^2^ Medtronic Inc. Mounds View Minnesota; ^3^ Department of Cardiology and Department of Anesthesiology and Perioperative Medicine The University of Texas MD Anderson Cancer Center Houston Texas USA

**Keywords:** proton therapy, pacemaker, defibrillator, dose perturbation

## Abstract

An increasing number of patients undergoing proton radiotherapy have cardiac implantable electrical devices (CIEDs). We recently encountered a situation in which a high‐voltage coil on a lead from an implanted cardiac defibrillator was located within the clinical treatment volume for a patient receiving proton radiotherapy for esophageal cancer. To study the effects of the lead on the dose delivery, we placed a high‐Z CIED lead at both the center and the distal edge of a clinical spread‐out Bragg peak (SOBP) in a water phantom, in both a stationary position and with the lead moving in a periodic pattern to simulate cardiorespiratory movement. We then calculated planned doses using a commercial proton treatment planning system (TPS), and compared them with the doses delivered in the phantom, measured using radiographic film. Dose profiles from TPS‐calculated and measured dose distributions showed large pertubrations in the delivered proton dose in the vicinity of the CIED lead when it was not moving. The TPS predicted perturbations up to 20% and measurements revealed perturbations up to 35%. However, the perturbations were less than 3% when the lead was moving. Greater dose perturbations were seen when the lead was placed at the distal edge of the SOBP than when it was placed in the center of the SOBP. We conclude that although cardiorespiratory motion of the lead mitigates some of the perturbations, the effects of the leads should be considered and steps taken to reduce these effects during the treatment planning process.

PACS numbers: 87.55.D‐,87.55.ne, 87.85.M

## I. INTRODUCTION

As the number of proton treatments has grown, so too has the number of proton‐treated patients who have cardiac implantable electrical devices (CIEDs), such as pacemakers and implanted cardioverter defibrillators (ICDs). Many studies have shown that X‐ray radiotherapy,^(^ ^1^ ^–^ ^4^ ^)^ computed tomography (CT) scans,^(^ ^5^ ^–^ ^6^ ^)^ and proton beam radiotherapy^(^ ^7^ ^)^ can have adverse effects on the operation of CIEDs including: generation of random defibrillation shocks when no shock is needed, inhibition of device operation resulting in the device not properly responding to adverse biological signals, and resetting or reprogramming of the device. These studies, along with recommendations and guidelines published by the American Association of Physicists in Medicine Task Group 34^(^ ^8^ ^)^ and the United States Food and Drug Administration,^(^ ^9^ ^)^ have led to the development of widely adopted treatment procedures to minimize the risk of these effects in patients with CIEDs. These procedures include keeping the device out of the radiation field, keeping the total dose to the device below manufacturer‐recommended levels, estimating the dose delivered to the device (using thermoluminescent dosimeters, metal‐oxide‐semiconductor field‐effect transistors, etc), and monitoring the function of the CIED on a daily or weekly basis.

However, even with these mitigating procedures, CIEDs could create problems during proton radiotherapy. Because of the coulombic nature of proton beam interactions with matter, the presence of multiple high‐Z materials (such as tantalum, titanium, and platinum) in the high‐voltage (HV) shock coils and pacing ring electrodes of the leads in CIEDs directly affects the delivered dose distribution. (To prevent confusion, it should be noted that ‘lead’ and ‘leads’ in this text always refers to an electrical component of the CIED and never the element represented by the symbol ‘Pb’.) This is known to be the case for other high‐Z materials that may be found in patients undergoing proton therapy. For instance, studies of the effects on dose distribution in high‐Z fiducial markers used for patient setup in radiotherapy for prostate cancer have reported dose perturbations ranging from 15% to 35% for stainless steel and titanium markers, depending on the size and orientation of the marker with respect to the treatment beam.^(^ ^10^ ^–^ ^12^ ^)^ Because many different lead arrangements (single‐chamber vs. multiple‐chamber placements) and types of leads with different geometries (e.g., standard pacing vs. ICD lead) are commonly used for CIEDs, it is important to understand the possible effects of these leads on the dose delivered during proton beam radiotherapy. To our knowledge, however, no studies have explored these effects.

We report a study of the perturbations to proton dose in the presence of an HV shock coil from an ICD lead within the clinical treatment volume (CTV) of a patient receiving proton radiotherapy for esophageal cancer. We investigated perturbations to delivered proton dose within a water phantom containing an HV ICD lead similar to the one implanted in the patient, using both measurements of delivered dose and calculations from a commercial proton therapy treatment planning system (TPS). On the basis of our study results, we briefly discuss the clinical implications of the presence of such leads within a proton radiotherapy treatment field and present some suggestions for accounting for the leads' effects during the treatment planning process.

## II. MATERIALS AND METHODS

### A.1 Patients

**Figure 1:**
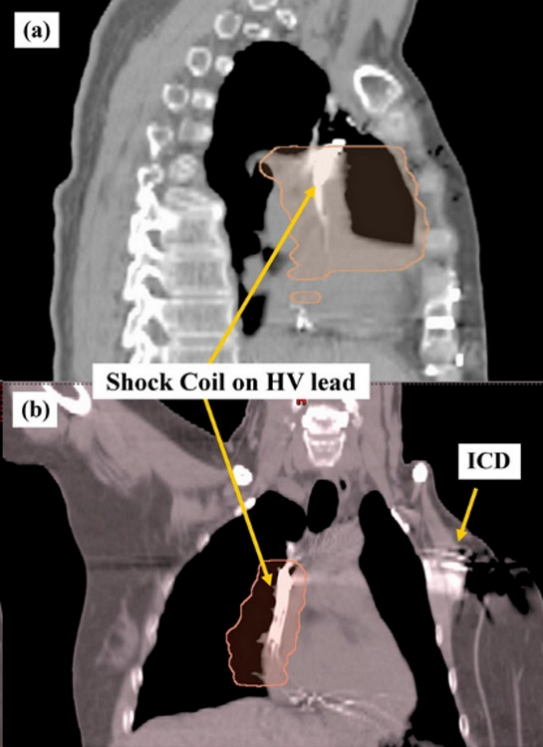
Sagittal (a) and coronal (b) computed tomographic images used in the treatment planning for a patient receiving proton radiotherapy for esophageal cancer. The patient's implanted cardioverter defibrillator (ICD) had a high‐voltage lead which had two pacing ring electrodes, a right ventricle shock coil, and a superior vena cava (SVC) shock coil, the last of which was located within the clinical treatment volume (CTV; pink shaded region).

We took CT scans during radiotherapy treatment planning for an esophageal cancer patient who had an ICD (Medtronic #6947, Medtronic Inc, Moundsview, MN). Figure 1 presents sagittal and coronal CT showing that although the ICD itself was not within the CTV, the HV shock coil of the lead, located in the superior vena cava, was well within it. The long, high‐CT number (CT#) artifact in the CT image was due to the ICD lead's HV shock coil, which was composed of tantalum, platinum, and iridium. The HV shock coil appeared to be much wider (~5 mm) in the CT images shown in Fig. 1 than it actually was (~2 mm diameter). This discrepancy was a result of movement of the lead within the surrounding anatomy during patient cardiorespiratory motion, as well as imaging artifacts caused by the high‐Z materials.

### A.2 Phantom study

**Figure 2:**
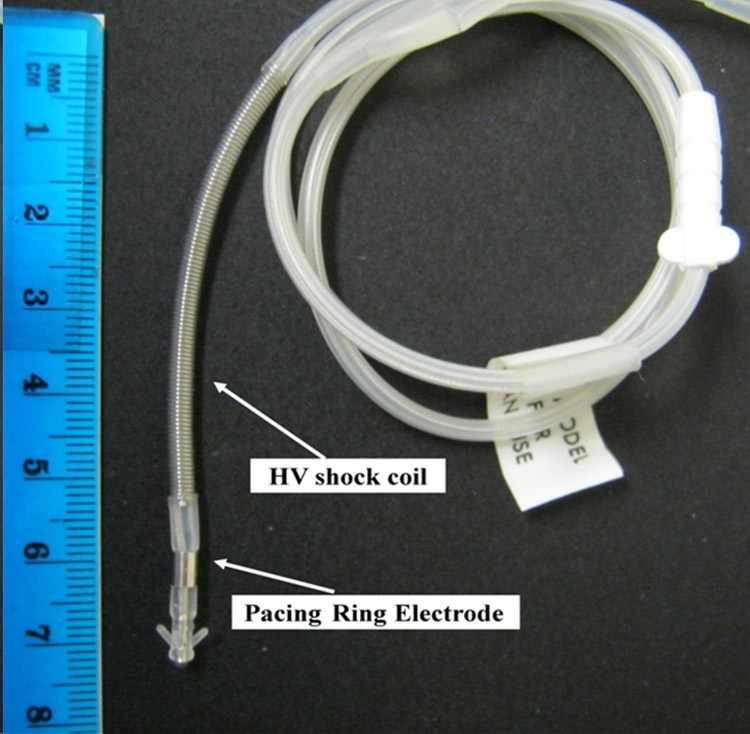
Implanted cardioverter defibrillator lead used in the phantom showing the shock coil and pacing ring electrodes of the ICD lead that were used to study dose perturbations to proton dose delivery.

**Figure 3:**
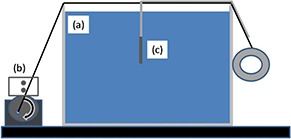
Schematic diagram of the periodically oscillating lead function water phantom (a) with an oscillating pulley system consisting of a string that is attached to the front rotating disk of a motion phantom (b), runs over the top of the water phantom, and is connected to a counterweight on the other side. The lead (c) is attached to the pulley string and hangs down into the water phantom. When the motion phantom is turned on, the lead harmonically oscillates from side to side with a range of motion of approximately 5 mm.

To determine the types and magnitude of dose perturbations caused by the presence of the HV shock coil, we conducted a phantom study with an ICD lead similar to the one in the patient (Medtronic model# 6932; Fig. 2) inserted into a water phantom. This lead contained an HV shock coil and pacing ring electrode similar to those of the lead implanted in the patient. A standard, single 200 MeV (same energy as used for patient treatment) clinical treatment beam incident on the front face of the phantom was used to deliver a uniform 200 cGy dose with a 10 cm × 10 cm treatment field and a 10 cm wide spread‐out Bragg peak (SOBP) with the lead positioned within the delivered treatment dose volume. The lead was placed in the water phantom at a depth along the beam path of 10.0 cm or 16.5 cm, representing the center and distal edge, respectively, of the SOBP delivered by the treatment beam (Fig. 3).

Additionally, we employed a Varian RPM motion phantom device (Varian Medical Systems, Palo Alto, CA) to create a periodically oscillating lead function (POLF) phantom that would mimic motion of the lead caused by breathing and cardiac motion. In the POLF phantom, the lead was attached to a pulley system composed of a nylon string attached at one end to the rotating disc of the motion phantom. The string ran over the top of the water phantom to the other side of the phantom, and a counterweight was attached to the string's other end (Fig. 3). This pulley system allowed the lead to move side to side in a periodically oscillating motion with a range of 5 mm (2.5 mm) in the plane lateral to the beam central axis, thus allowing us to model the motion of the lead seen in the patient CT images.

Before delivering the proton beam, we performed four‐dimensional (4D) CT scans of the POLF phantom using a GE LightSpeed RT16 CT scanner (General Electric, Fairfield, CT). These scans were conducted first with no lead present in the phantom and then with the lead placed at each depth studied (10.0 cm and 16.5 cm), with periodic motion and with no motion of the lead. The no‐motion scan was performed by disconnecting the pulley string from the rotating phantom wheel and fixing the lead position at the center of the range of motion produced by the POLF phantom pulley system. The full 4D datasets were sorted into 10 respiratory phases (0%–90%), and full cine and average CT datasets were created.^(^ ^13^ ^–^ ^14^ ^)^

After acquiring the 4D CT scans with each phantom‐lead setup, the CT images were imported into a Varian Eclipse (version 8.9, dose algorithm: proton_ 8908a) TPS, and a simple, single‐field treatment plan was designed. The treatment was first planned using CT images of the POLF phantom without the lead present and then copied onto the CT image sets containing the lead, after which the dose was recalculated (with all beam delivery parameters fixed) in the presence of the lead. This mimics the clinical method for treatment planning when high‐Z materials are present — high‐Z artifacts are overridden to tissue density during planning, the override is then removed and the dose recalculated.

The treatment plan was used for each setup: lead at the center of the SOBP with no motion, lead at the center of the SOBP with motion, lead at the distal edge of the SOBP with no motion, and lead at the center edge of the SOBP with motion. For each of the setups, the lead was located approximately 5 cm from the lateral edge of the planned treatment field. This treatment plan contained no patient‐specific beam range compensator (to correct for the presence of high‐density materials in the beam path^(^ ^15^ ^)^ so that the full potential effect of the lead on dose distribution could be evaluated.

As the 200 cGy beam was delivered to the POLF phantom for each setup, measurements of the delivered dose were made using GAFCHROMIC EBT2 dosimetry film (International Specialty Products, Wayne, NJ). Films were positioned in the water phantom perpendicular to the beam, 2 mm behind the lead at both the bottom tip of the lead and near the top of the lead (2.5 cm superior to the tip). These positions corresponded to the position of the pacing ring electrode of the lead (bottom tip) and the lead's HV shock coil (top of lead). The exposed films were scanned using a commercial Epson 10000 XL flatbed scanner (Epson America, Long Beach, CA), and a cubic polynomial fit calibration curve was used to convert the data from optical density to dose. Full details of the procedure used to analyze the irradiated films were published by Ciangaru et al.^(^ ^16^ ^)^ Each irradiated film was scanned 3 times, and the values from each scan were averaged together to give the final reported measured values for each setup, resulting in a 1 sigma uncertainty in the measured values of less than 2%.

**Figure 4:**
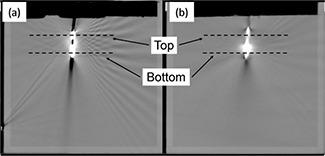
Computed tomographic images of the lead in the periodically oscillating lead function phantom showing (a) no motion and (b) 5 mm motion of the lead. Film was placed parallel to the plane of the CT slice immediately distal to the lead. The axis of the proton beam was normal to the plane of the CT slice. Dashed lines indicate the location beneath the surface of the water at which 1D lateral cross‐field profiles were extracted from behind the high‐voltage shock coil electrode (Top) and from behind the pacing ring electrode (Bottom).

**Figure 5:**
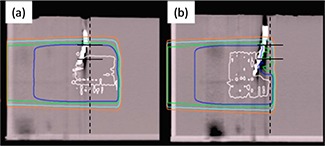
Treatment planning system (TPS) calculations of dose deposition. Shown are depth dose profiles with the lead located near (a) the center of the spread‐out Bragg peak (SOBP) and (b) the distal edge of the SOBP (no motion in both positions). The normalized dose distribution is shown with the isodose lines representing the 100% (white), 90% (dark blue), 80% (green), 50% (light blue), and 25% (orange) dose levels; dashed vertical lines indicate the position behind the lead at which films were positioned for measurement and the depth along the beam path at which lateral profiles were extracted from TPS calculations; solid horizontal lines through the dashed lines indicate where on the film the lateral profiles were sampled.

Using the averaged values of the scanned film images, one‐dimensional (1D) lateral dose profiles along the direction perpendicular to the beam central axis at both the top and bottom of the lead were extracted from each film. The locations of the measured 1D dose profiles are indicated in Fig. 4 (depth beneath the water surface) and Fig. 5 (distal to the HV lead). The measured 1D dose profiles were compared to 1D dose profiles extracted from the same positions in the TPS‐calculated dose distribution (Fig. 5).

## III. RESULTS

CT scans of the ICD lead in the POLF phantom revealed that the lead produced significant CT artifacts (see Fig. 4). These included a large, high‐CT# artifact caused by the lead's pacing ring electrode and shock coil, with a number of noticeable artifacts radiating outward from the lead. The spatial extent of the high‐CT# artifact that was caused by the lead was somewhat smaller in CT scans of the no‐motion setups than in scans of the motion setups. However, the magnitude of the CT#s for pixels within the artifacts (that is, the severity of the artifacts) was much higher for the no‐motion setups (CT#s 2800–3071) than for the motion setups (CT#s 1225–2600). This is because the CT#s for the moving lead were averaged over all phases of the 4D CT scan in the average CT dataset. The CT#s measured for the motion setups in the POLF phantom were in good agreement with those measured from the patient CT dataset (CT#s 1400–2750), indicating that the oscillating lead motion in the phantom gave a reasonable estimation of the effects of cardiorespiratory motion on the appearance of the lead in the CT datasets.

Calculations of dose delivery made with the TPS indicate that the pacing ring electrode and shock coil can cause significant perturbations to the delivered dose distributions. In discussing these results, ‘range’ will be used to mean the physical depth to the distal 80% dose (unless otherwise stated) rather than the water‐equivalent path length of the beam. As shown in Fig. 5, with no lead motion, the presence of the lead in the center of the SOBP caused a decrease of approximately 5 mm in the range of the treatment beam. However, with the lead placed near the distal edge of the SOBP, the proton beam range was degraded more, by up to 30 mm, with the location of the distal 90% range of the SOBP occurring within the lead. The difference in range perturbations between the setups occurred because the average energy of the protons interacting with the lead placed near the distal SOBP edge was much lower than the energy of protons interacting with the lead in the center of the SOBP. Because low‐energy protons interact and deposit energy at a faster rate than high‐energy protons,^(^ ^17^ ^)^ the high‐Z materials in the shock coil and the pacing ring electrode that interacted with the protons caused greater proton scattering and energy loss near the distal edge of the SOBP than at shallower depths within the SOBP.

**Figure 6:**
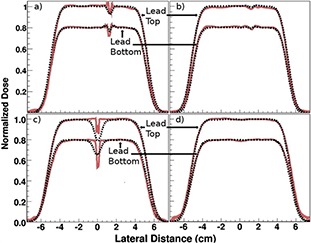
Lateral dose profiles at the top and bottom of the lead (as indicated in Fig. 4) extracted from treatment planning system‐calculated (dashed black line) and film‐measured (solid red line) dose distributions: (a) lead at spread‐out Bragg peak (SOBP) center with no motion; (b) lead at SOBP center with 5 mm motion; (c) lead at SOBP distal edge with no motion; and (d) lead at SOBP distal edge with 5 mm motion. Note: Profiles through the lead top and bottom are normalized to the average dose in the flat region of the dose profile, and then the normalized lead bottom data is multiplied by 0.8 to distinguish it from the lead top profile for display purposes only.

Lateral 1D dose profiles taken from both TPS calculations and film measurements of the delivered doses for all setups are shown in Fig. 6. For the setup with the lead in the center of the SOBP and no motion of the lead (Fig. 6(a)), the 1D lateral dose profile at 2 mm behind the top of the lead extracted from the TPS‐calculated dose distribution revealed the presence of a cold spot with a 7% decrease in dose relative to the average dose in the flat region of the profile, with a full width at half‐maximum width (FWHM) of 5 mm. The 1D dose profile at the top of the lead extracted from the measured dose distribution (from the film) showed a cold spot with an 8% dose decrease and a FWHM of 3 mm. Additionally, this profile contained a noticeable dose enhancement of approximately 5% on either side of the cold spot.

For the same setup (SOBP center, no motion), the 1D dose profile at 2 mm behind the bottom of the lead extracted from the TPS‐calculated dose distribution showed a cold spot with a 4% dose decrease and a FWHM of approximately 2 mm. The profile extracted from the measured dose distribution showed a cold spot with a 10% dose decrease and a FWHM of 3 mm. At the bottom of the lead, profiles from both the TPS‐calculated and measured dose distributions showed dose enhancements of approximately 3% on either side of the cold spot.

In the setup with the lead at the center of the SOBP and 5 mm periodic motion of the lead in the POLF phantom (Fig. 6(b)), the effects of the lead on dose distribution were greatly reduced, if not completely washed out, compared with the no‐motion setup. The dose profile at the top of the lead extracted from the TPS‐calculated dose distribution showed a dose decrease of approximately 2% flanked by a slight increase on either side, and the profile from the measured dose distribution showed a 3% dose decrease.

Profiles at the bottom of the lead (again, SOBP center with motion) from the TPS‐calculated dose distribution also showed a dose decrease of approximately 2% flanked by a slight increase on either side, and those taken from measured dose distributions showed a cold spot with a 3% dose decrease.

For the setup with the ICD lead at the distal end of the SOBP and no motion of the lead (Fig. 6(c)), the 1D dose profile at the top of the lead from the TPS‐calculated dose distribution showed a 20% dose decrease with a FWHM of 9 mm. The profile from the measured dose distribution contained two sharp dose decreases of 14% and 12% on the edges of the cold spot, with an average dose decrease of 10% in the center of the cold spot. This pattern, with a larger dose decrease at the edges of the cold spot than in the center, was created by the structure of the shock coil, which had a thin wire wrapped around the lead's outer edges. The overall FWHM of the cold spot was 3 mm on the profile taken from the measured dose distribution.

For the same setup (SOBP distal end, no motion), the 1D dose profile at the bottom of the lead taken from the TPS‐calculated dose distribution showed a cold spot of 18% with a half‐maximum width of 9 mm, and the profile taken from the measured dose distribution showed a cold spot of 35% with a FWHM of 3 mm.

Finally, with the lead at the distal end of the SOBP and 5 mm periodic motion of the lead (Fig. 6(d)), no significant dose decrease was present in the dose profiles taken from either the TPS‐calculated or measured dose distributions at either the top or bottom of the lead.

## IV. DISCUSSION

Our results showed that, given the presence of several high‐Z materials in the shock coils and pacing ring electrodes of leads in CIEDs, significant cold spots and small hot spots in the dose distribution can occur behind the leads. Dose perturbations were more severe when the leads were positioned closer to the distal edge of the SOBP, with cold spots of up to 35% and reductions of the beam range of up to 30 mm immediately behind the lead. These results are similar to those of Newhauser et al.,^(^ ^11^ ^)^ who demonstrated that clinically relevant dose perturbations were caused by high‐Z, implanted fiducial markers used in proton therapy for prostate cancer.

On the basis of our results, we conclude that the effects of CIED leads on dose distribution should be accounted for if the leads are present within the path of the treatment beam during proton radiotherapy, especially if a lead shock coil or pacing ring electrode must be included in the CTV. In such cases, we recommend overriding the CT# for the CIED lead in the patient CT scans used for treatment planning with the CT# seen with no motion present (i.e., the CT# from a single phase of the 4D CT or possibly from the maximum‐intensity‐projection CT dataset). This will ensure that dose calculations performed by the TPS provide a “worst‐case scenario” of treatment dose perturbation, allowing a conservative treatment plan to be developed that accounts for the maximum possible effect of CIED leads. If possible, it is best to choose beam angles to minimize the path of the beam through the CIED lead. In general the beam will be perpendicular to the lead (the lead typically being oriented in the superior–inferior direction), and the path through the CIED will not vary much as a function of beam angle. Additionally, it is best to avoid plans that have the lead at the distal edge of the treatment field, as this scenario produced the greatest dose perturbations.

It should be noted that for our phantom study, the lead moved independent of the phantom material (water) behind the lead. However, in a clinical setting, the lead is implanted within and attached to the surrounding tissue (i.e., the interior wall of the superior vena cava; see Fig. 1), which means that the lead moves in conjunction with the tissue to which it is attached. Thus, in a clinical setting, the tissues located immediately behind the HV shock coil and pacing ring electrode, with respect to the beam, will most likely stay in the shadow of these structures as the lead moves with cardiorespiratory motion. Therefore, even though our results showed that the effects of the lead on delivered dose were mitigated by the motion, the dose effects that occurred in the no‐motion treatment may be clinically applicable because the tissue behind the lead would be attached to the lead and, therefore, would most likely stay in the shadow of the lead during dose delivery. Our recommendation to override the CT# of the lead in the patient scan used for planning to the CT# seen without motion is motivated by and accommodates this possibility. In fact, because we did not measure tissue motion with respect to the motion of the lead, the actual dose perturbations encountered clinically may lie somewhere between our findings for the 5 mm motion treatment and the no‐motion treatment.

Additionally, we note that the simple treatment plan we used in our study did not include a range compensator to correct for inhomogeneous tissue densities in the path of the beam. The use of a range compensator will help account for the high‐Z materials in the CIED lead, thus reducing perturbations to the delivered dose. However, because of the small size of the different electrodes on these leads (~2 mm in diameter), any slight misalignment of the patient or changes in the motion of the lead (e.g., because of changes in respiration) would reduce the effectiveness of the compensator to correct for the lead and possibly introduce new perturbations in the delivered dose.

Even though the specific results (i.e., the magnitude of dose perturbations) presented in this paper may pertain only to the specific lead design studied, we believe that the types of perturbations (cold spots and dose enhancements) encountered in this study will be typical of those seen for any HV CIED lead (defibrillator and possibly standard pacing leads) located within the path of a treatment beam during proton beam radiotherapy. With the wide variability in both commonly used CIED lead designs and CT scanning techniques used for treatment planning simulation, the appropriate CT#s should be determined on a case‐by‐case basis. We cannot recommend the use of CT#s quoted in this paper for situations involving different CIED lead designs that may be encountered in practice.

## V. CONCLUSIONS

The presence of a CIED lead in the clinical treatment volume can result in clinically relevant perturbations of the delivered dose in proton radiotherapy, with the greatest effects seen when the lead is near the distal edge of the proton beam range. Our study showed perturbations that could be as high as 35% if a CIED lead is at the distal edge of the SOBP. Even though cardiorespiratory motion of the lead mitigates some of the perturbations, the effects of the leads should be considered and steps should be taken to reduce these effects during the treatment planning process.

## ACKNOWLEDGMENTS

This work was conducted in collaboration with Medtronic Inc. The University of Texas MD Anderson Cancer Center is supported in part by a Cancer Center Support Grant (CA016672) from the National Institutes of Health.
